# Supplementary material for: Speckle rheological spectroscopy reveals wideband viscoelastic spectra of biological tissues
Source: bioRxiv. 2023 Jun 9:2023.06.08.544037. Preprint. [Version 1] doi: 10.1101/2023.06.08.544037 (PMC10274797; doi:10.1101/2023.06.08.544037)
Supplement: Supplement 1 [file media-1.pdf]

Speckle rheological spectroscopy reveals wideband viscoelastic spectra of biological tissues: supplementary information

Nichaluk Leartprapun[1], Ziqian Zeng[1], Zeinab Hajjarian[1], Veerle Bossuyt[2], & Seemantini K. Nadkarni[1,*]

[1]Wellman Center for Photomedicine, Massachusetts General Hospital, Harvard Medical School, Boston, MA 02114 USA
[2]Department of Pathology, Massachusetts General Hospital, Boston, MA 02114 USA
*Corresponding author: snadkarni@mgh.harvard.edu

Content

Supplementary Table 1. Wideband spectroscopic parameters of fibrin constructs, whole blood clots, breast tissues, and bones

Supplementary Table 2. Optical properties and scattering particle size of fibrin constructs, whole blood clots, breast tissues, and bones

Supplementary Table 1. Wideband spectroscopic parameters of fibrin constructs, whole blood clots, breast tissues, and bones. Spectroscopic parameters extracted from frequency-dependent $|G^*(\omega)|$, $G'(\omega)$, $G''(\omega)$, and $\alpha(\omega)$ spectra measured by wideband SHEARS. Unless otherwise specified, values of G and ω are reported in units of kPa and rad/s, respectively. Values indicate mean±standard deviation of N=9 (fibrin construct), 5 (whole blood clot), and 3 (breast tissue and bone) measurements.

**Parame-ters**	**Fibrin construct**	**Whole blood clot**		**Breast tissue**							**Bone**	
		**High FIB**	**Low FIB**	**Fibrous**	**Adipose**	**Fibrous tumour stroma**			**Tumour epithelium**		**Cortical**	**Trabecular**
	(Fig. 1d)	(Fig. 2a)	(Fig. 2b)	(Fig. 3b)	(Fig. 3e)	(Fig. 3d)	(Fig. 3g)	(Fig. 3i)	(Fig. 3j)	(Fig. 3h)	(Fig. 4b)	(Fig. 4c)
Regime I												
ω_T	20 ± 3	0.36 ± 0.09	-	13 ± 4	100 ± 30	30 ± 10	18 ± 3	16 ± 2	20 ± 10	8 ± 7	30 ± 20	23 ± 7
Regime II												
ω^0_{II}	300 ± 100	3.1 ± 0.7	0.8 ± 0.2	210 ± 20	1500 ± 200	100 ± 20	86 ± 3	100 ± 30	100 ± 40	50 ± 20	1720 ± 70	140 ± 20
G^0_{II}	0.7 ± 0.2	0.6 ± 0.2 Pa	0.3 ± 0.2 Pa	3.5 ± 0.6	1.27 ± 0.06	4.6 ± 0.9	0.90 ± 0.09	0.61 ± 0.05	500 ± 100	0.3 ± 0.2	5000 ± 2000	300 ± 100
Regime III												
γ_{IIIa}	0.4 ± 0.1	0.60 ± 0.01	0.68 ± 0.05	0.20 ± 0.03	0.8 ± 0.1	0.33 ± 0.09	0.44 ± 0.03	0.4 ± 0.1	0.45 ± 0.02	0.62 ± 0.03	0.56 ± 0.09	0.1 ± 0.1
α_{IIIa}	-	-	-	-	0.44 ± 0.05	0.22 ± 0.06	0.27 ± 0.05	0.25 ± 0.05	0.40 ± 0.01	-	-	0.16 ± 0.03
$\omega_{T,III}$	-	110 ± 50	40 ± 30	-	-	-	-	-	-	-	-	-
ω_γ	2420 ± 70	160 ± 30	200 ± 30	2000 ± 200	-	-	-	-	-	900 ± 200	-	-
γ_{IIIb}	1.2 ± 0.1	0.83 ± 0.01	0.80 ± 0.02	0.73 ± 0.05	-	-	-	-	-	0.51 ± 0.09	-	-
α_{IIIb}	0.50 ± 0.09	0.83 ± 0.01	0.80 ± 0.02	0.33 ± 0.05	-	-	-	-	-	0.51 ± 0.09	-	-
Regime IV												
$\omega^0_{IV}\times10^3$	-	-	-	200 ± 100	-	2.3 ± 0.7	3.1 ± 0.3	2 ± 1	3.3 ± 0.4	-	90 ± 30	1.5 ± 0.2
G^0_{IV}	-	-	-	13 ± 2	-	8 ± 2	2.1 ± 0.4	1.2 ± 0.6	1.7 ± 0.4	-	6000 ± 2000	500 ± 200
Regime V												
γ_V	-	-	-	0.5 ± 0.1	-	0.55 ± 0.06	0.58 ± 0.06	0.3 ± 0.2	0.7 ± 0.1	-	0.2 ± 0.2	0.78 ± 0.06
α_V	-	-	-	-	-	0.26 ± 0.01	0.28 ± 0.07	0.26 ± 0.06	0.45 ± 0.07	-	-	0.44 ± 0.07
Regime VI												
$\omega^0_{VI}\times10^3$	-	-	-	-	-	130 ± 40	90 ± 30	-	-	-	-	-
G^0_{VI}	-	-	-	-	-	18 ± 5	5 ± 2	-	-	-	-	-
Regime VII												
γ_{VII}	-	-	-	-	-	0.6 ± 0.3	0.35 ± 0.01	-	-	-	-	-

Supplementary Table 2. Optical properties and scattering particle size of fibrin constructs, whole blood clots, breast tissues, and bones. Estimated material parameters used in the reconstruction of frequency-dependent $G^*(\omega)$ as detailed in Methods. μ_s': reduced scattering coefficient, μ_a: absorption coefficient, a: sphere-equivalent hydrodynamic radius.

	**Optical properties**	**Particle radius, *a***
**Fibrin construct** (Fig. 1)	μ_s' 0.6 mm^{-1}	0.18 µm
**Whole blood clot** (Fig. 2)	μ_s' 2.1 mm^{-1}, μ_a 0.1 mm^{-1}	2.88 µm[†]
**Breast tissue** (Fig. 3)	μ_s' 1.5–3.2 mm^{-1}*	0.1 µm[††]
**Bone** (Fig. 4)	μ_s' 4.8 mm^{-1} (cortical)	12 nm[†††]
	μ_s' 3.5 mm^{-1} (trabecular)	

[†]Based on the hydrodynamic size of RBC[1].
[††]Based on prior study in breast cancer specimens[2].
[†††]Based on the crystal size of bone minerals[3].
*The fibrous stroma tends to be more highly scattering than the cellular regions.

References

1 Friebel, M., Roggan, A., Müller, G. & Meinke, M. Determination of optical properties of human blood in the spectral range 250 to 1100 nm using Monte Carlo simulations with hematocrit-dependent effective scattering phase functions. *J. Biomed. Opt.* **11**, 034021 (2006).

2 Hajjarian, Z., Brachtel, E. F., Tshikudi, D. M. & Nadkarni, S. K. Mapping Mechanical Properties of the Tumor Microenvironment by Laser Speckle Rheological Microscopy. *Cancer Res.* **81**, 4874-4885 (2021).

3 Turunen, M. J. *et al.* Bone mineral crystal size and organization vary across mature rat bone cortex. *J. Struct. Biol.* **195**, 337-344 (2016).
